# The Importance of Starting With Goals in N-of-1 Studies

**DOI:** 10.3389/fdgth.2020.00003

**Published:** 2020-05-22

**Authors:** Sean A. Munson, Jessica Schroeder, Ravi Karkar, Julie A. Kientz, Chia-Fang Chung, James Fogarty

**Affiliations:** 1 Human Centered Design and Engineering, DUB Group, University of Washington, Seattle, WA, United States,; 2 Computer Science and Engineering, DUB Group, University of Washington, Seattle, WA, United States,; 3 Informatics, Indiana University Bloomington, Bloomington, IN, United States

**Keywords:** N-of-1, goals, patient-centered, self-tracking, self-monitoring, self-experiment

## Abstract

N-of-1 tools offer the potential to support people in monitoring health and identifying individualized health management strategies. We argue that elicitation of individualized goals and customization of tracking to support those goals are a critical yet under-studied and under-supported aspect of self-tracking. We review examples of self-tracking from across a range of chronic conditions and self-tracking designs (e.g., self-monitoring, correlation analyses, self-experimentation). Together, these examples show how failure to elicit goals can lead to ineffective tracking routines, breakdowns in collaboration (e.g., between patients and providers, among families), increased burdens, and even designs that encourage behaviors counter to a person’s goals. We discuss potential techniques for eliciting and refining goals, scaffolding an appropriate tracking routine based on those goals, and presenting results in ways that advance individual goals while preserving individual agency. We then describe open challenges, including how to reconcile competing goals and support evolution of goals over time.

## INTRODUCTION

N-of-1 designs have received attention for their potential to facilitate understanding and management of health conditions that require individualized insights and approaches (1, 2). N-of-1 studies come in a variety of designs [e.g., Heyvaert and Onghena (3) and Daskalova et al., (4)], including self-monitoring, in which people track data related to their condition to examine progress toward goals or changes over time [e.g., Mishra et al., (5), Ayobi et al., (6), and Consolvo et al., (7)]; correlational analyses, in which people investigate what factors may affect their symptoms [e.g., behavioral, environmental, medical; (8)]; and self-experiments, in which people determine causality between factors and symptoms [e.g., Karkar et al., (9) and Riggare et al., (10)]. Each of these designs can support a range of goals. Self-monitoring can support tracking and tuning behaviors and understanding whether a condition is worsening (providing cues to take action) or improving (providing motivation to continue one’s current management plan). Correlational analyses can support diagnosis and formation of hypotheses among possible contextual and behavioral factors and resulting outcomes. Self-experiments can provide additional rigor in testing relationships between individualized contributors and symptoms and in evaluating whether a management plan is effective.

Many people, individually or with support and encouragement from their healthcare providers, begin n-of-1 studies but struggle to achieve their goals (11–13). Drawing on research on using technology to collect, integrate, and reflect on data about oneself [self-tracking or personal informatics; (14)], we first examine how designers of n-of-1 tools often take a “data-first” perspective that does not place sufficient emphasis on understanding and supporting personalized goals. We then illustrate how this perspective leads to misalignments between people’s goals and the tools they use, or among people collaborating to understand and manage a health condition, as well as emergent design techniques for addressing these challenges. Finally, we discuss unaddressed challenges for researchers and designers of tools that support n-of-1 studies.

## ARTICLE CONTEXT: OUR RESEARCH IN N-OF-1 STUDIES AND TOOLS

In this viewpoint article, we examine and reflect on findings from our n-of-1 research. Across this research, we have surveyed 1,396 people with chronic conditions, and conducted interviews, participatory design sessions, or focus groups with 108 people with chronic conditions and 32 health providers (9, 15–21). We also draw on three field deployments of novel prototype systems with 48 people (22, 23). Although most these studies were grounded in specific conditions (irritable bowel syndrome, migraine, juvenile idiopathic arthritis) or health behaviors (sleep, healthy eating), we anticipate the implications of this research apply broadly to n-of-1 studies. All studies were approved by the Human Subjects Division at the University of Washington.

### Hypothesis Formation and Hypothesis Testing in Irritable Bowel Syndrome Management

Irritable bowel syndrome (IBS) is characterized by episodic gastrointestinal symptoms that are often caused by individualized dietary factors [i.e., different nutrients can be “triggers” for different individuals; (24)]. Providers often advise their patients record their foods and symptoms in a journal to attempt to identify these triggers, but both patients and providers struggle to interpret the data (16). We explored how the design of n-of-1 tools can better address these challenges. We then examined how interactive, exploratory visualizations can help people and their health providers better interpret their data and collaborate with each other (17). We further developed Foodprint, a photo-based food journaling system that reduces burden and explicitly elicits the patient’s goals to better support personalized, actionable, collaborative review (23). Finally, we examined how self-experimentation could help people determine causality between a symptom and trigger (22). We designed, developed, and evaluated a system to support such self-experimentation, and investigated how Bayesian analyses could better answer the questions people want to answer via self-experiments (20). Together, these studies provide insights around how n-of-1 tools can help people form and test hypotheses about their personal IBS triggers.

### Supporting Distinct Personalized Goals in Migraine Management

Migraine is characterized by unpredictable, intermittent, and poorly understood symptoms. Similar to IBS, providers often recommend their patients with migraine self-track to better understand and manage their migraines, but both again struggle to find value in the resulting data (18). We investigated how to better support individualized migraine management. We first investigated challenges and pitfalls people currently face, characterizing distinct types of migraine tracking goals people would like to pursue. We then developed and investigated *goal-directed self-tracking*, a new method that scaffolds the process of deciding what, when, and how to track toward a specific goal, and analyzes and visualizes the resulting data to support that goal (20).

## SOURCES AND CONSEQUENCES OF GOAL MISALIGNMENTS

Both the tools used for n-of-1 studies and the people involved in planning, interpreting, and acting on those studies can be sources of goal misalignments. We draw on our results, as well as related research, to illustrate these misalignments.

### Tools as Source of Goal Misalignment

People conducting n-of-1 studies for insights into their health often adopt tools that are misaligned with their personal goals. These misalignments generally fall into three categories: (1) designs that operationalize a broad goal in ways that are inconsistent with an individual’s operationalization of that goal; (2) assumptions that a tracking goal implies other long-term goals; (3) data-first views that fail to scaffold use of that data to support individualized goals.

Designers often make assumptions about how people pursuing their own n-of-1 studies operationalize their goals in their daily lives. For example, many applications designed to support healthy eating promote calorie-counting goals (15). People pursue healthy eating goals, and adopt tools in support of those goals, for much more varied reasons (e.g., improving their energy levels, adopting a diet that they believe has health benefits, trying to reduce certain foods, managing an eating disorder) (15, 25). Tools that operationalize every goal as calorie counting both fail to support people’s true goals and can lead to negative feelings and counterproductive behaviors (15).

Similarly, designers often assume that people track to pursue certain long-term goals. For example, most commercial menstrual tracking apps embed an assumption that people track to become or avoid becoming pregnant (26). This assumption can lead to features that are irrelevant or hurtful (e.g., an annoyance for people who are not having sex that could result in conception, a painful reminder for people who cannot conceive). We have been happy to see a trend toward making such features optional and disabled by default (e.g., in Apple’s new cycle tracking application), but more work is needed to apply such design principles consistently across self-tracking tools.

An approach of creating general-purpose tools that allow people to collect a large range of data in various ways and to run analyses on that data may seem promising; a flexible tool could support a range of goals (27). However, this approach also leads to problems. Some self-tracking applications *do* enable collection and integration of large amounts of data, with the idea that supporting flexibility is the same as supporting individualized goals. However, *flexibility* is not the same as *support*. Added flexibility for configuration requires a system to also support understanding *how* to configure for one’s goals. This problem is particularly salient in n-of-1 tools, where people may not know what goals are achievable or reasonable (20) or how to translate their goals into tracking plans (28).

Even when people bring their own well-defined, achievable goals to n-of-1 tools, they face burdens when tracking and analyzing resulting data. They may reach incorrect conclusions or abandon a tool after considerable effort but without reaching their goals (11, 29). Tracking tools designed with a data-first view may also prioritize collection of as much data as possible, even when lower-burden tracking would also support a person’s goals.

### People as a Source of Goal Misalignment

Whether people conducting n-of-1 studies initiate those studies themselves or under the advice of a health provider, they frequently turn to others for support (e.g., family, peers, health experts). However, collaborators sometimes assume certain goals for both for *why* the person is tracking (e.g., *management goals* regarding what they want to address in their health and *tracking goals* regarding the information can help them achieve those management goals) and *how* they should track. Such assumptions can introduce misalignment in configuring, interpreting, and acting on self-tracking data (19).

#### Misaligned Management Goals

A person’s goals for managing their health sometimes differs from their health provider’s (16). For example, when reviewing food and symptom diaries, providers often try to identify potential contributors to a patient’s digestive symptoms and suggest they eliminate those potential contributors. However, due to personal preferences and priorities, patients may instead choose to continue eating certain foods, tolerating resulting symptoms and planning for how those symptoms will affect their lives. Other patients may initially restrict their diet as suggested to control their symptoms, then collect food and symptom data toward a goal of re-diversifying their diet, which a provider may not expect if not explicitly told (17). Patients also sometimes use food and symptom diaries to elicit emotional support, such as seeking recognition of their effort in managing symptoms or showing the data as evidence of how symptoms affect their life. Although providers may primarily expect to use data for diagnosis and the design of treatment plans, acknowledging these other potential patient goals is also important throughout the collaboration.

People with migraine and health providers also encounter tensions when their management goals do not align (18). For example, prescription medications can prevent symptoms for some people with migraine. As many providers assume their patient’s primary management goal is symptom prevention, a common first step in migraine treatment is to prescribe medications. However, some people with migraine have management goals of preventing symptoms *without* medications. One patient described this misalignment: “[My doctor’s] approach was much more like, ‘Let me figure out what drugs I can give you to have you stop having these headaches’, rather than figuring out why I’m having them. I’m much more like, ‘I want to know why this is happening’.”

#### Misaligned Tracking Goals

Even when management goals do align, misalignment in tracking goals can still be detrimental in collaborations (e.g., within a family, between patients and providers). When providers encourage patients to track what they eat and relevant health indicators, they sometimes review the tracked data with the patient once and then expect the patient to continue independently reviewing data. However, provider disengagement with data can dissuade people from continuing tracking or suggest that self-tracking is no longer useful. We found similar misalignments in migraine, where some providers assumed patients would be able to analyze their data to identify trends.

Providers can also be removed from the lived experience of self-tracking, leading them to recommend burdensome tracking routines (16, 20). For example, providers might assume patients want the most validated answers possible and recommend rigorous but high-burden tracking (e.g., paper diaries detailing every consumed food). Patients may instead sacrifice some rigor to find a tracking regime that fits better in their life (e.g., a photo-based food diary that loses some detail but retains a reasonable record with less effort). In migraine, providers do not always recognize the burden of daily tracking, so they might recommend it given potential value of having more data [e.g., “obviously I like my patients to track every day,” (20)]. However, people might prefer to reduce their tracking burden by building in breaks or tracking only when they experience symptoms.

Misalignments in management and tracking goals also interact to create further problems. For example, many people with migraine track with a goal of predicting the likelihood of symptoms so they can prepare for or attempt to prevent those symptoms. They often focus on tracking contributors to ensure they will notice contributor accumulation, which can result in symptoms. However, providers generally focus on overall symptom frequency, rather than the consequences of symptoms on a particular day. They therefore often want patients to focus on tracking treatments and symptoms. A patient’s desired tracking routine may therefore differ from their provider recommendations.

## BETTER SUPPORT FOR GOALS IN N-OF-1 TOOLS AND PROCESSES

Emerging design patterns can support explicit goal alignment and pursuit. These patterns include supporting patients and providers in aligning goals, configuring tracking routines to support goals, and analyzing and presenting resulting data to provide actionable insights that advance goals.

### Eliciting and Aligning Patient and Provider Goals

Systems and health experts can prompt patients to articulate their goals, which can help people plan their tracking and subsequent actions.

**FIGURE 1 |:**
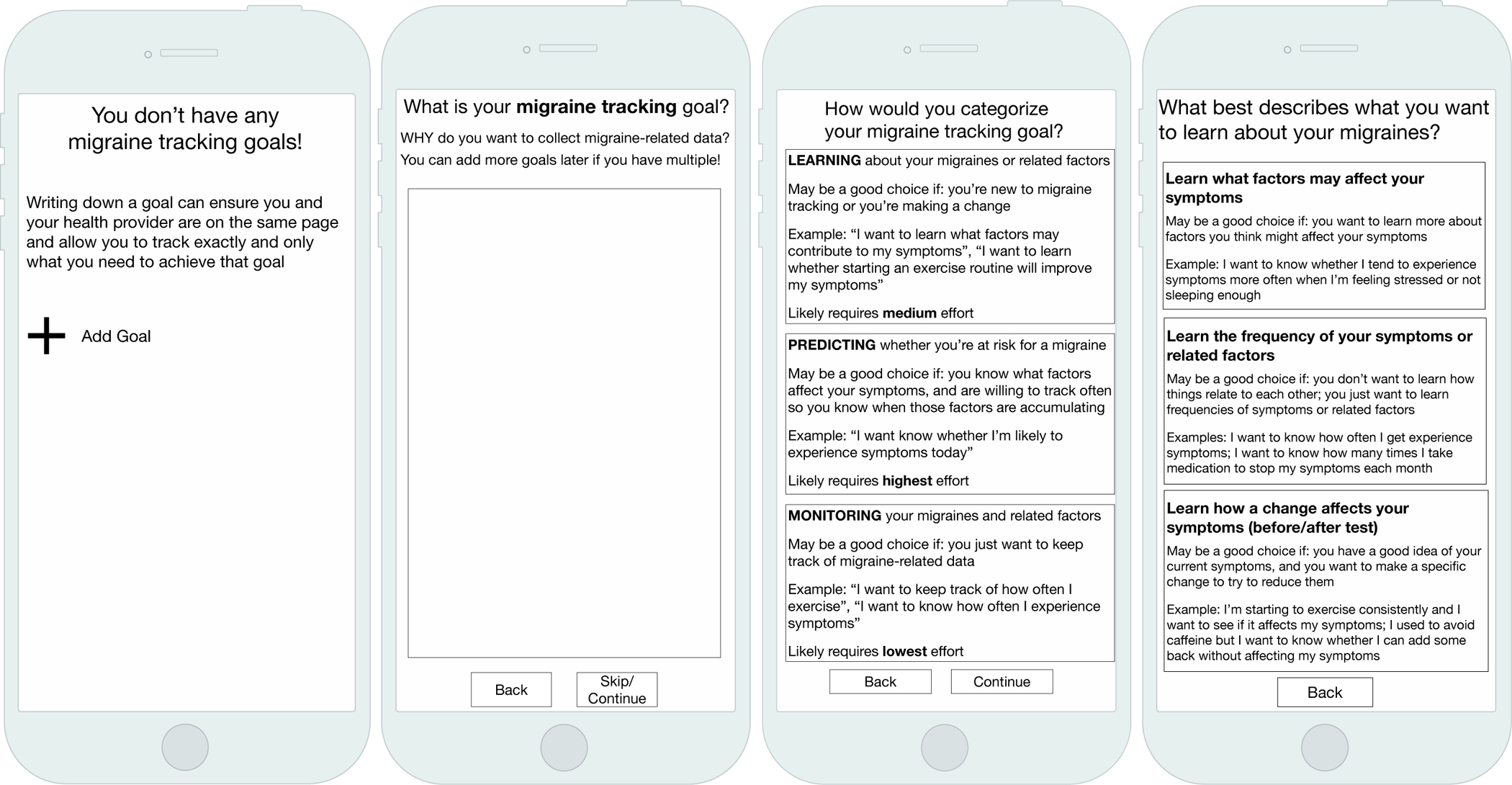
Our goal-directed self-tracking prototype for migraine elicited people’s tracking and management goals.

In our research to support n-of-1 studies in migraine, we designed an interface to elicit a person’s tracking goals (Figure 1). We first asked people to define their migraine tracking goal in their own words. Participants sometimes struggled to express their goals, but presenting explicit examples helped them reconcile their management and tracking goals. After expressing their goal, our design prompted people to categorize that goal as one of three distinct migraine tracking goal categories we had previously characterized (18). All participants reported being able to select a goal category that articulated why they wanted to track migraine-related data. The explicit categories also helped participants hypothesize about goals they might want to pursue in the future and helped differentiate those goals from their current goals. For example, one participant wanted to focus on learning about her migraines, but thought that, once she understood more, she would want to transition to a monitoring or predicting goal. Another ultimately wanted to learn about her migraines but decided to first focus on a lower-burden monitoring goal before committing to a goal that might require longer or more frequent tracking routines. Explicit goal categories therefore helped participants to navigate the critical path between their management and tracking goals and to reason about what goals would be most feasible and helpful to them at present and in the future.

Designs can also support communication about goals between patients and providers. In our food tracking research, we designed a pre-visit note to support explicit patient-provider communication about goals (23). The note elicited patient goals for the visit, their own summary of their data, and their questions for health providers. Providers could view this note at the start of a visit with that patient. Both patients and providers paid more attention to the patient’s goals and questions during visits with the note. Having these explicit goals also helped providers tailor their advice to patient priorities. For example, one provider saw that a patient valued eating certain foods that could contribute to symptoms. Rather than urging that patient to eliminate those foods, they instead talked about alternative ways to prepare them that might mitigate symptoms. Another patient-provider pair also chose to focus on stress management instead of food elimination, because the patient had a goal of maintaining dietary diversity. Having awareness of a patient’s goal allowed providers to better develop individualized management plans.

### Scaffolding the Right N-of-1 Study Design Based on a Person’s Goals

After a person’s goals are understood, designs can scaffold n-of-1 studies that support those goals with the least burden by either matching people with the right tool among many or by changing how a tool is configured.

**FIGURE 2 |:**
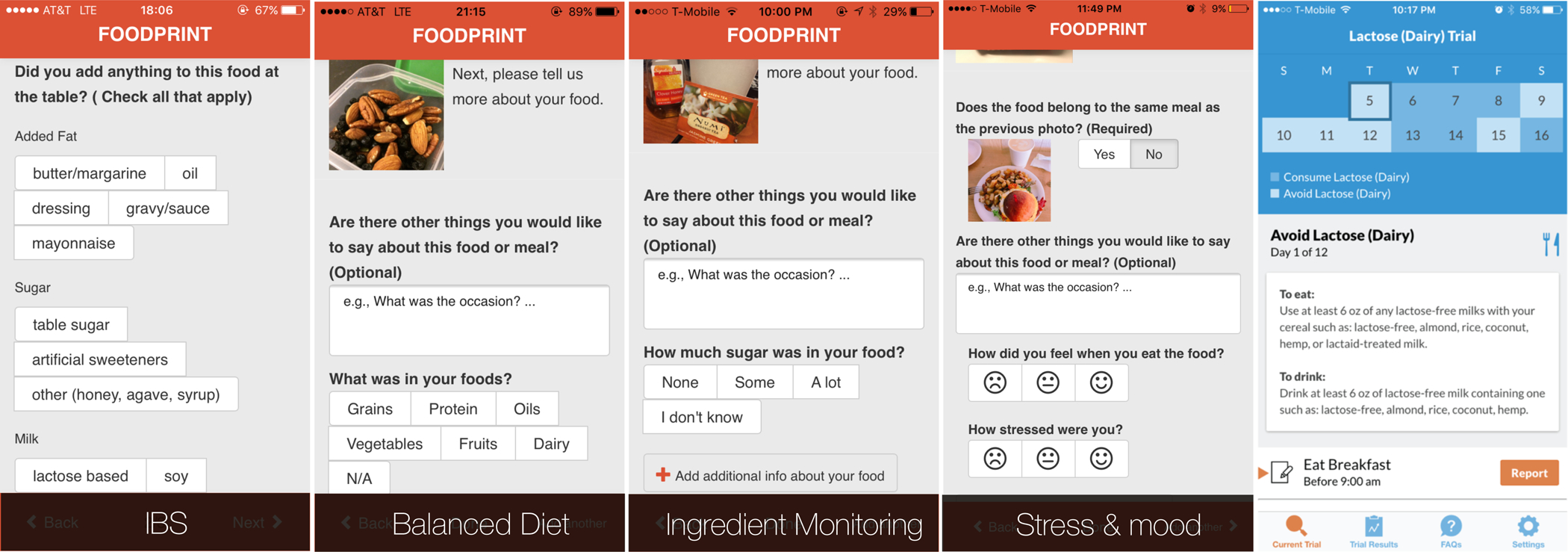
By eliciting people’s tracking goals, we could configure the Foodprint food diary application to better support those goals. People who wanted to test the relationship between a specific potential contributor and symptoms, however, benefited more from using our self-experimentation application, TummyTrials (rightmost).

To support healthy eating and IBS management, Foodprint supported configurations specific to different goals (23) (Figure 2, left). For example, when individuals expressed a healthy eating goal of “eating more balanced meals,” we configured their app to support annotating food groups (fruits, vegetables, grains, protein, dairy, oils). Individuals who wanted to understand relationships between food and mood or stress could instead report stress level and mood. Finally, for people tracking to understand potential IBS symptom contributors, we configured Foodprint to record common contributors and symptoms. During onboarding, researchers elicited patient goals to configure the tool, but we envision the design of onboarding processes that elicit goals and configure an appropriate n-of-1 tracking tool.

In our work on goal-directed self-tracking for migraine, we designed and evaluated low-fidelity prototypes for such an interface (20). Transforming goals into tracking regimes helped people avoid common tracking pitfalls. The system could prompt people to track all of the data they would need to support their goal, avoiding a breakdown in which people do not track all the data needed to meet their goals. It also could guide them away from tracking *too much* data, avoiding a breakdown in which people track too much, become fatigued, and abandon tracking. For example, when a person selects a goal of *identifying contributors to their migraines*, the system walks them through selecting symptoms and contributors they want to investigate. When a person selects a goal of *monitoring* their migraines, which typically does not require tracking contributors, the system encourages a focus on symptoms.

Given the variety of possible management and tracking goals, no single tool can realistically support every goal a person might have. Tool *selection*, and communication of a tool’s limits, is therefore as important as tool *configuration*. Consider a person working to understand what factors contribute to their gastrointestinal symptoms. They might use Foodprint for preliminary analyses that suggest caffeine or lactose may be a trigger. However, that person might only consume caffeine when stressed, and might only consume caffeine in lattes. Each of these factors (i.e., caffeine, lactose, and stress) is a potential contributor, but Foodprint’s correlational approach cannot untangle their confounds. Doing so requires a rigorous self-experiment, which is challenging for people to design and conduct due to the need for expertise in health, experimental design, and appropriate tracking burden (11). A system designed to scaffold such self-experiments (Figure 2, right) can design an appropriate experiment and explicitly support a corresponding tracking routine and analyses of results (9, 22). Guiding people to the right tool for their goal is therefore necessary: a person with a specific hypothesis would likely prefer a self-experimentation app, whereas a person who wants to learn about potential contributors would likely prefer a tool that supports correlation-based analyses of a broader range of factors.

Each n-of-1 design has a range of possible analysis approaches. In our correlational analysis of food photos, we explored both visual analysis of photos grouped by symptom severity and quantitative analysis graphing correlations between nutrients and symptoms (17, 23). Both approaches had advantages. Photos facilitated conversation and better supported action planning; the quantitative analysis supported understanding more complex nutrient-symptom interactions. Our work in self-experimentation revealed other tradeoffs. Our initial analysis presented a graph and a summary of a statistical analysis, including a *p*-value (22). This familiar (although flawed) statistical detail contributed to a sense of validity and trust for participants. We have since shown that Bayesian analyses can better support the questions people ask from n-of-1 studies and the decisions they want to make using those answers (18). Across all study designs and analyses, grounding results in examples from a person’s data (e.g., particular foods, days when symptoms were severe) facilitated understanding and helped them determine next steps.

## FUTURE CHALLENGES

Our research shows that eliciting goals and configuring systems to support them is often less straightforward than it sounds. Participants often approach tracking with underspecified [e.g., “I don’t know [what my goal is]. I just want to know how to get rid of them faster,” (20)] or unachievable goals. Techniques outlined above can help, but goal elicitation and specification remain challenging (28, 30).

Even when people can articulate and fulfill tracking goals, knowing the answer to a question can be far from acting on it. Research should develop techniques for providing actionable guidance tailored to a person’s goals and their context, such by using explicit goal elicitation alongside context-aware computing [e.g., Rabbi et al., (31) and Lee et al., (32)]. Similar to our scaffolding for migraine tracking (20), others have proposed interactive instructional materials, such as for effective planning (32). Designers might also develop techniques for helping people anticipate possible answers to a range of possible questions, so they could decide whether they would want to act on any of those answers *before* they begin tracking. This information would allow people to exclude n-of-1 studies designed to provide answers on which they would not want to act or that would provide insufficient evidence for them to act.

Fully supporting goal-directed self-tracking also requires supporting goal evolution, both between and within goals. People often change their goals as their understanding, experiences, and needs change (19, 33–35). For example, a person with migraine may initially want to learn about their migraines (e.g., understand what causes their symptoms), then switch to monitoring. Tools should support explicitly making such changes.

We have thus far designed and evaluated n-of-1 systems that focus on one person’s goals and what their health providers believe those goals are or should be. However, many health behaviors are influenced by others, especially the people with whom one cohabitates, such as family members (36, 37). In such situations, we might instead think of the unit of analysis as a family. Within that family, people might have shared goals, compatible goals, or conflicting goals (21). Such uses will likely require new n-of-1 designs and approaches.

## CONCLUSION

New technologies for collecting, integrating, and analyzing data promise to make n-of-1 studies more feasible than ever before. This trend offers important opportunities for understanding and managing personal health. However, we caution against assumptions, and especially implicit assumptions, about why and how people use tracking tools. Such assumptions often lead to frustrating goal misalignments and n-of-1 studies that provide the wrong answers or no answers. Instead we urge researchers and designers to start with people’s goals, then provide scaffolding to support selection and configuration of tools to meet those goals.
